# Application of Marine Microbial Natural Products in Cosmetics

**DOI:** 10.3389/fmicb.2022.892505

**Published:** 2022-05-26

**Authors:** Jinwang Ding, Baochuan Wu, Liqun Chen

**Affiliations:** ^1^Institute of Applied Genomics, Fuzhou University, Fuzhou, China; ^2^College of Biological Science and Engineering, Fuzhou University, Fuzhou, China

**Keywords:** marine microbes, natural products, cosmetic applications, metagenomic technology, biotransformation

## Abstract

As the market size of the cosmetics industry increases, the safety and effectiveness of new products face higher requirements. The marine environment selects for species of micro-organisms with metabolic pathways and adaptation mechanisms different from those of terrestrial organisms, resulting in their natural products exhibiting unique structures, high diversity, and significant biological activities. Natural products are usually safe and non-polluting. Therefore, considerable effort has been devoted to searching for cosmetic ingredients that are effective, safe, and natural for marine micro-organisms. However, marine micro-organisms can be difficult, or impossible, to culture because of their special environmental requirements. Metagenomics technology can help to solve this problem. Moreover, using marine species to produce more green and environmentally friendly products through biotransformation has become a new choice for cosmetic manufacturers. In this study, the natural products of marine micro-organisms are reviewed and evaluated with respect to various cosmetic applications.

## Introduction

The skin is the largest organ of the human body and plays an important protective role (Resende et al., 2021). As humans age, their skin becomes thinner and loses its original elasticity and moisturizing ability. Aged skin is dry, flabby, wrinkled, and increasingly fragile (Wang et al., 2015; Resende et al., 2021). Because skin has prolonged contact with the outside world, external factors, such as UV radiation, dust, and chemical reagents, can reduce skin’s antioxidant capacity and accelerate its aging rate (Resende et al., 2021). Skin care is essential for maintaining its appearance and health, but it also strengthens the barrier function of the skin (Bedoux et al., 2014). The concept of skin care is well known. With the idea of skincare gaining popularity, many antiaging creams, moisturizers, and sunscreens are on the market. However, the majority of cosmetics sold are composed of synthetic chemicals, which may have harmful side effects (Morais et al., 2021). Parabens, the most widely used preservatives in cosmetics, have been reported to mimic the effects of estrogen, increase the risk of breast cancer in women, and influence the development of malignant melanoma (Kerdudo et al., 2016). The California Department of Environment has found that chemicals in cosmetics and personal care products are associated with birth defects in male reproductive organs, reduced sperm count, and altered pregnancy outcomes in animal experiments (Barrett, 2005). Although the study did not show the same effect in humans, it was met with serious skepticism when it was published. Safety-conscious consumers are no longer looking for skin cosmetics that only provide fragrance and temporary adornment, but are willing to pay more for cosmetics containing natural ingredients that are more beneficial to the skin (Draelos, 2018). It is estimated that the global market value of natural cosmetics will reach USD 54.5 billion by 2027 (Thiyagarasaiyar et al., 2020). With the continuous increase in consumer demand and the expansion of the cosmetics market, it is necessary to develop a large number of active natural substances. Interestingly, the ocean attracts developers because of its unique environment (high pressure, high salt, and low temperature). Shu Uemura’s first addition of seawater to skin-care products has led researchers to explore the use of marine natural products in cosmetics (Martins et al., 2014).

Finding novel active natural compounds is the main target of developing new cosmetics. The oceans cover more than 70% of the Earth’s surface. They are the largest habitat on Earth and are home to 90% of all living organisms (Gomez et al., 2009; Dash et al., 2012). Therefore, marine micro-organisms are considered important potential sources of active natural products. Some bioactive compounds from marine micro-organisms have antitumor activity (Rehman et al., 2020), anti-inflammatory activity (Toledo et al., 2014), and antibacterial activity (Nalini et al., 2018). The natural products of marine microbes have received great attention in the cosmetics industry. However, few active compounds derived from marine micro-organisms have been used in the cosmetics industry. In this paper, we reviewed the application and possible mechanism of active substances derived from marine micro-organisms in sun protection, whitening, moisturizing, anti-aging, repair, etc. (Supplementary Table 1). In addition, some possible difficulties and solutions of natural products in the cosmetics industry derived from marine micro-organisms are discussed to provide a reference for the cosmetics industry.

## Sunscreen

Part of the UV radiation emitted by the sun is absorbed by oxygen molecules in the stratosphere (UVC, 100–290 nm), part is absorbed by the ozone layer (UVB, 290–320 nm), and the remainder of UVA radiation (UVA, 320–400 nm) and a small part of UVB are transmitted to the ground (Battistin et al., 2020). Basking in the sun can help the conversion of 7-dehydrocholesterol into vitamin D in human skin, but long-term exposure to UV light causes mutagenic and non-specific damage to the epidermis. UV-induced reactive oxygen species (ROS) can alter genetic material and inhibit the production of extracellular matrix proteins. This can contribute to the loss of skin elasticity, skin photoaging, actinic keratosis, and skin cancer (Guillerme et al., 2017; Battistin et al., 2020; He et al., 2021).

**Figure 1:**
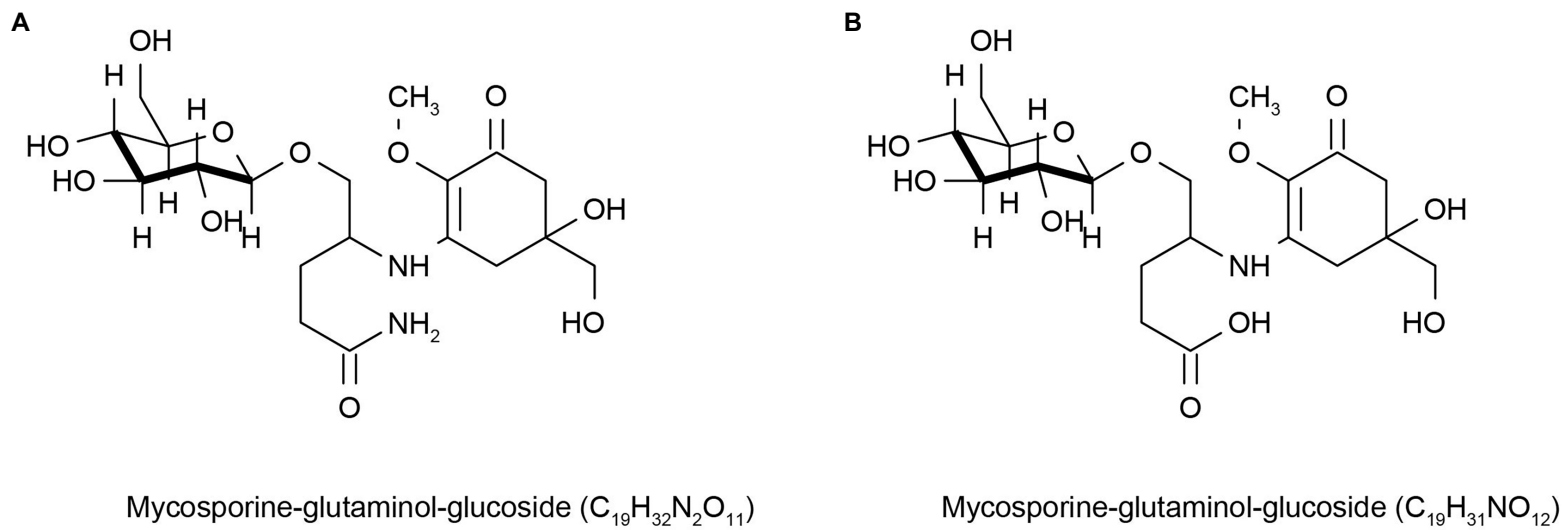
Reprinted with the kind permission of Environmental Chemistry publications. Chemical structures of Mycosporine-glutaminol-glucoside **(A,B)** isolated from fungi hypersaline waters and polar glacial ice. Adapted from Kogej et al. (2006).

Sunscreens are a sun protection products that help reduce the damage caused by UV rays, but some sunscreens lack light stability, irritate the skin, and cause allergic reactions (Greenspoon et al., 2013). In addition, these compounds can also affect marine life (Downs et al., 2015) and the marine environment (Sang and Leung, 2016; Chaves Lopes et al., 2020) when discharged into the ocean with sewage. Therefore, the cosmetics market is constantly looking for new environmentally friendly UV-resistant molecules to change this situation. Lower eukaryotes, such as marine microalgae, have evolved mechanisms to synthesize secondary metabolites unrelated to their growth and reproduction, but most of these metabolites can interact with UV light to coordinate cell functions (Kostyuk et al., 2018). This led to widespread concern in the cosmetics market. Many marine microbial sources of mycosporine-like amino acids (MAAs) can absorb UV rays ranging from 310 to 360 nm. They are colorless, water-soluble, and low molecular weight compounds composed of cyclohexanone or cyclohexanol imine chromophores with the nitrogen substituent of an amino acid or its imino alcohol, which inhabit fungi, algae, cyanobacteria, and other marine organisms. MAAs are also considered a potential source of environmentally friendly sunscreen ingredients with good anti-UV activity (Sinha et al., 2007; Sun et al., 2020; Nedeljka, 2021). People’s interest in MAAs has increased in recent years. MAAs are distributed in the cytoplasm of fungal cells and can also be released into extracellular colony mucus, enhancing UV protection for themselves and possibly for other community members (Llewellyn and Airs, 2010). Kogej et al. (2006) found two different UV-absorbable mycosporine-glutaminol-glucosides (Figure 1) and three unidentified UV-absorbable compounds in fungi in glaciers and high-salt waters, which they considered to be MAAs. These are natural sources of MAAs, which will have a good market value if they can be further applied to cosmetics. Some sunscreens with MAAs as the main active ingredient have been marketed globally, such as Helionori® and Helioguard365® (Chrapusta et al., 2017). However, some microbes are difficult to grow in laboratory conditions because of environmental constraints, which impede access to these metabolites. Fortunately, the current sequencing technology is very advanced, and people can obtain the relevant gene cluster information of the metabolites of MAAs produced by marine micro-organisms based on metagenomic sequencing technology, obtain the genes through the chemical synthesis *in vitro*, and then transform them into *Escherichia coli* for production using genetic engineering. Miyamoto et al. (2014) found gene clusters for MAAs synthesis in *Actinosynnema mirum* DSM 43827 and *Pseudonocardia* P1. These genes were not expressed or rarely expressed, in laboratory culture conditions. However, silenced MAAs gene clusters were expressed in the *Streptomyces* sp. *avermitilis* SUKA22 strain after genetic engineering. This result encourages the cosmetics industry to obtain these natural active products and helps accelerate the innovation and development of the cosmetics industry.

Moreover, carotenoids, such as astaxanthin, zeaxanthin, lycopene, and β-carotene are the most common pigments in nature and have biological functions such as light capture and photoprotection (Vílchez et al., 2011). Microalgae are important sources of carotenoids, fatty acids, and amino acids (Ambati et al., 2018). *Haematococcus lacustris* (formerly *Haematococcus pluvialis*; Chlorophyta) can accumulate astaxanthin under high salinity, high temperatures, and light stress (Naguib, 2000). This microalga has the highest astaxanthin yield and has great application potential (Kuang et al., 2019). Astaxanthin has a higher antioxidant activity than lutein, lycopene, β-carotene, and other carotenoids (Naguib, 2000). The antioxidant activity of astaxanthin is 10 times that of zeaxanthin, lutein, and β-carotene (Matsuno and Miki, 1990). This is due to the carbonyl functional groups in the ionone ring of astaxanthin (Liu and Osawa, 2007). Due to its strong antioxidant activity, astaxanthin has been added as its active ingredient in some cosmetics brands around the world, such as Geisha Astaxanthin Refining Serum, Home Facial Pro Astaxanthin Essence, and Kose Becky Firming Essence (Kuang et al., 2019). However, astaxanthin is unstable and prone to oxidation and discoloration, so better technologies are needed to develop and expand astaxanthin applications. Zeaxanthin is an important factor in the photoprotection mechanism of *Nannochloropsis oculata* (Shen et al., 2011). Microalgal-derived fucoxanthin protects against sunburn (Matsui et al., 2016). Lycopene neutralizes oxygen free radicals and is a powerful natural antioxidant with the potential to be used as a sunscreen ingredient (Mourelle et al., 2017). Hashtroudi and colleagues isolated strains that produce carotenoids from Iranian terrestrial and aquatic ecosystems. *Anabaena* (Cyanobacteria) was the first time a strain with the highest natural lycopene production was identified (Hashtroudi et al., 2013). In addition to the microalgae, scytonemin in the outer sheath of cyanobacteria mainly absorbs UVA radiation, while MAAs are keys to protecting against UVB radiation. At low MAA-content conditions, the carotenoids in cells are rapidly synthesized and bind to the outer membrane to act as photoprotection (Ehling-Schulz et al., 1997). Scytonemin is a unique natural product that consists of indole and phenolic subunit dimers. It can be used in sunscreens due to its anti-UV and antioxidant effects (Mourelle et al., 2017; Stoyneva-Gärtner et al., 2020). Additionally, cyanobacteria have high carotenoid potential (Mezzomo and Ferreira, 2016). All in all, carotenoids produced by cyanobacteria and microalgae have great potential for use in cosmetics.

**Figure 2:**
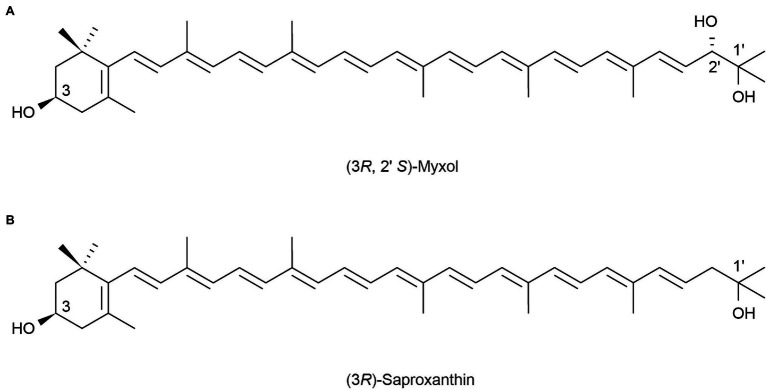
Reprinted with the kind permission of Applied Microbiology and Biotechnology publications. Chemical structures of two rare monocyclic carotenoids (3R, 2’S)-Myxol **(A)** and (3R)-Saproxanthin **(B)**. Adapted from Shindo et al. (2007).

**Figure 3:**
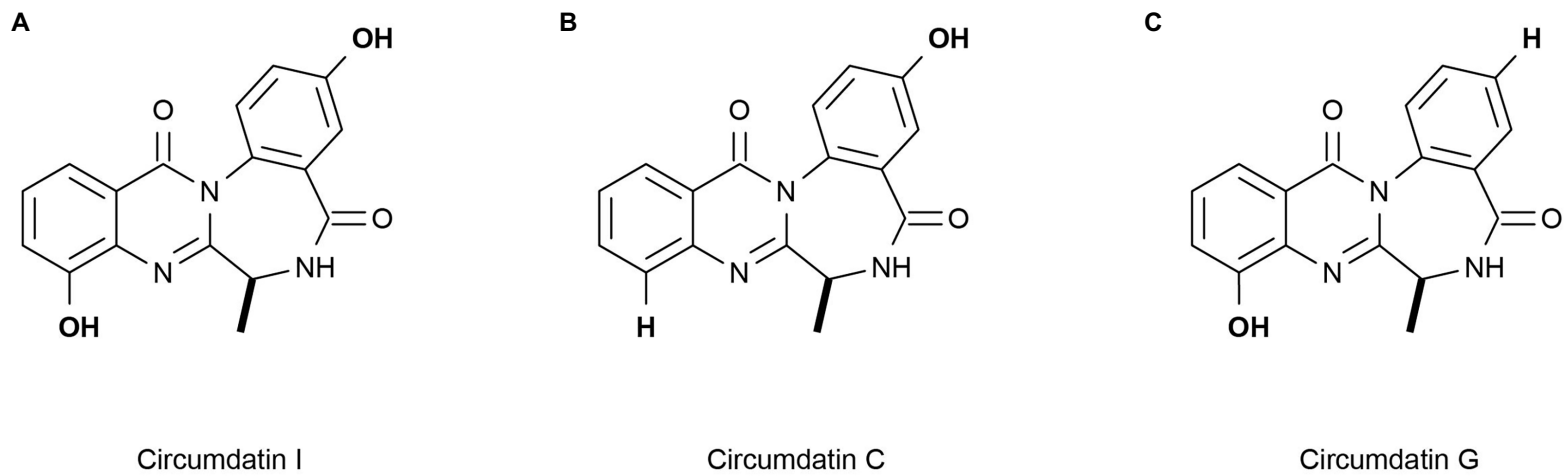
Reprinted with the kind permission of The Journal of Antibiotics publications. Chemical structures of Circumdatin I **(A)**, C **(B)**, and G **(C)**. Adapted from Zhang et al. (2008).

Marine heterotrophic bacteria and fungi are also potentially important sources of carotenoids. *Phaffia*, *Rhodozyma*, and *Xanthophyllomyces* produce large amounts of astaxanthin (Ambati et al., 2014). Two rare, monocyclic carotenoids, (3*R*, 2’*S*)-myxol (Figure 2A) and (3R)-saproxanthin (Figure 2B), were found in a new bacterium species from the family Flavobacteriaceae isolated in Okinawa, Japan. Compared to zeaxanthin and β-carotene, saproxanthin and myxol showed stronger antioxidant activity (Shindo et al., 2007). Zhang et al. (2008) isolated a new benzodiazepine alkaloid, cyclosporine I (Figure 3A), from the metabolites of the marine fungus *Exophiala*. This compound had a structure similar to cyclosporine C and G (Figures 3B,C) from the same source and had good anti-UVA activity. Compared with microalgae, the amount of carotenoid produced by marine fungi and bacteria is small, but fungi and bacteria are easy to culture and rapidly reproduce. Thus, they can be modified using genome sequencing and genetic engineering to improve carotenoid production (Galasso et al., 2017). These findings suggest that there may be other exploitable substances in marine micro-organisms with anti-UV activity.

Natural ingredients can provide protection through the direct absorption of UV rays and protect skin from UV damage through antioxidant action (He et al., 2021). Many antioxidants derived from marine organisms have been studied for their UV protection. Two compounds, a golmaenone of the diketopiperazine alkaloid and neoechinulin A of related alkaloids, were isolated from the marine fungus *Aspergillus* sp. They showed the activity of scavenging free radicals against 1, 1-diphenyl 2-picrylhydrazyl (DPPH) and showed better anti-UV activity than oxybenzone in sunscreen (Li et al., 2004). Most lipophilic vitamins also have strong antioxidant activity (Lourenço-Lopes et al., 2020). Tocopherol (vitamin E) is only synthesized by photosynthetic organisms and has the same antioxidant activity as carotenoids. Tocopherol was studied in 130 cultured microalgae and cyanobacteria [including 118 microalgal strains from four phylogenetic lineages (Chlorophyta, Streptophyta, Heterokontophyta, and Rhodophyta) and 12 cyanobacterial strains] by Mudimu et al. (2017). They found that α-tocopherol was most abundant, while β-tocopherol and γ-tocopherol were present in some algae but in lower amounts. Among them, the α-tocopherol production of Chlorophyta was higher than that of Rhodophyta, which could be a natural source of α-tocopherol (Mudimu et al., 2017). Sivakumar et al. (2014) discovered a new strain (*Stichococcus bacillaris* strain siva2011) that produces natural RRR-α-tocopherol and could be commercialized. Leya et al. (2009) found two strains of *Raphidonema* from snow and permafrost substrates, which were good α-tocopherol producers (Leya et al., 2009). Shanuja et al. (2018) extracted pigment from a marine *Aspergillus nidulans* and found that it had structural similarity to 5, 6-dihydroxyindole-2-carboxylic acid (DHICA), the precursor of melanin. It can reduce ROS generation after UV irradiation and has the potential for use in sunscreen formulations.

Moreover, some other natural molecules have anti-UV activity, such as phenolic substances. Phenolic compounds are produced during the production of ROS and are considered stress-induced compounds with protective mechanisms against UV radiation and anti-reactive oxygen (Bedoux et al., 2014). Phenols also play an important role in the antioxidant mechanism of cells and research on phenols in marine algae has been conducted (Stoyneva-Gärtner et al., 2020). Haoujar and colleagues tested four microalgae: *Phaeodactylum tricornutum* (Bacillariophyta), *Microchloropsis gaditana* (formerly *Nannochloropsis gaditana*; Ochrophyta, Eustigmatophyceae), *Nannochloris* sp., and *Tetraselmis* (Chlorophyta), and found that the antioxidant capacity of microalgae was positively correlated with the content of phenolic substances. *Phaeodactylum tricornutum* had the highest phenolic content and the best antioxidant activity among the four microalgae. These results indicate that *P. tricornutum* is a potential source for developing novel antioxidant substances (Haoujar et al., 2019). Duval et al. (1999) isolated *Chlorella* sp. (Chlorophyta) from a snow microalgal community at King George Island and showed that the total content of phenols in *Chlorella* increased under UVA and UVC irradiation. This confirms that phenols contained in microalgae act as antioxidants when stimulated by UV light (Haoujar et al., 2019). Although algae are rich in phenolic compounds, it has been found that the content of phenolic compounds in algae extracts also depends on the algae collection site and extraction method (Mateos et al., 2020). This requires conducting topographic analysis and selecting extraction methods before development to maximize the use of phenolic substances in algae.

## Whitening Effect

Skin, consisting of the epidermis and dermis, plays a protective role in the human body. The epidermis is the outermost layer of the skin and is mainly composed of melanocytes and keratinocytes. Melanocytes provide melanin to keratinocytes through the dendritic transfer of melanin bodies so that keratinocytes can form melanin caps and reduce UV-induced DNA damage to the epidermis (Costin and Hearing, 2007). One’s skin color is mainly determined by the amount, type, and distribution of melanin in the skin (Dessinioti et al., 2009). There are two types of melanin, eumelanin (dark brown insoluble polymers) and non-melanin (reddish sulfur-containing polymers). Tyrosinase is an important enzyme in melanin synthesis (Alves et al., 2020). Under its catalysis, tyrosine can be converted into dopaquinone, which is finally converted into melanin through a series of complex processes (Dessinioti et al., 2009; Agrawal et al., 2018). Marine organisms are important sources of compounds that can inhibit tyrosinase. The kojic acid produced by the fungi *Altenaria* sp. isolated from the surface of marine *Ulva lactuca* (Chlorophyta) has been shown to have tyrosinase activity (Li et al., 2003). Li et al. (2005) isolated *Myrothecium* sp. (Fungi) strain MFA58 from marine green algae. Two cyclopentenone compounds, designated as myrothenones A and B, were found in this strain. Only myrothenone A showed tyrosinase inhibitor activity and its activity was greater than that of kojic acid, which is currently used in sunscreen products. Tsuchiya et al. (2008) found that homothallin II produced by marine *Trichoderma viride* (Fungi) may inhibit tyrosinase activity by competing for the copper ion active site. Wu et al. (2013) isolated two new sesquiterpene compounds, 1β, 5α, 6α, 14-tetraacetoxy-9α-benzoyloxy-7βH-eudesman-2β, 11-diol and 4α, 5α-diacetoxy-9α-benzoyloxy-7βH-eudesman-1β, 2β, 11, 14-tetraol, from the marine fungus *Pestalotiopsis* sp. strain Z233, which also showed tyrosinase inhibitory activity. The *Micromonospora* sp. strain SH-89, which is symbiotic with sponges, also showed significant inhibitory activity against tyrosinase, as described by Said Hassane et al. (2020). Some substances produced by marine organisms that inhibit tyrosinase activity have been used commercially, but other substances (hydroquinone) have been banned in all European countries as they threaten human health (Burger et al., 2016). Therefore, there is a constant search for new, active whitening molecules. Marine micro-organisms such as microalgae and bacteria can also produce active substances with whitening functions. Astaxanthin, produced by marine yeast, has been found to protect skin from age spots (Corinaldesi et al., 2017). Astaxanthin and zeaxanthin produced by microalgae from *Nannochloropsis oculata* (Ochrophyta, Eustigmatophyceae) or *H. lacustris* (Chlorophyta) have antityrosinase activity (Balasubramaniam et al., 2021). Some whitening active substances derived from marine micro-organisms have also been discovered. A N-acyl dehydrotyrosine derivative derived from *Thalassotalea* sp. (Bacteria) strain PP2-459 isolated from crustaceans can act as a tyrosinase inhibitor superior to commercial kojic acid and arbutin (Deering et al., 2016). Cell extracts with the activity of cell-free tyrosinase were produced in a *Pseudomonas* sp. isolated from the waters off Ganghwa Island, South Korea. An extract of dichloromethane from the secondary metabolite of these new bacteria can reduce the melanin deposition in cultured skin and zebrafish. This extract has a whitening effect and could be used as a novel whitening ingredient (Kang et al., 2011). Kim et al. (2017) isolated the *Bacillus* strain SCO 147 from Gwangyang Bay, South Korea. The (−)-4-hydroxysattabacin metabolite from its crude extract had an anti-melanogenic effect in a human epidermal model and they identified it as a new natural melanin reducing agent (Kim et al., 2017). Khan et al. (2021) found that cydromicin (1), a secondary metabolite produced by the *Tolypocladium* sp. (Fungi) strain SCSIO 40433 isolated from arctic glacial sediments, also showed tyrosinase inhibitor activity. It follows that polar fungi are also potential sources of natural active substances. Pseudoalteromone A (1), a ubiquinone derivative produced by APmarine002 and RoA-050 strains of *Pseudoalteromonas* sp. (Bacteria) of marine origin, can inhibit tyrosinase activity by inhibiting melanin-producing gene expression. The whitening effect of the substance was evaluated by using a 3D pigment epidermis model, and it was confirmed that the substance had whitening and brightening effect, which provided a new source of active molecules for whitening products in cosmetics (Lim et al., 2021).

## Moisturizing Effect

The main cell type of the dermis is the fibroblast, which is embedded in collagen, elastic fibers, and an extracellular matrix composed of glycoprotein, hyaluronic acid, glycosaminoglycan, and other mixtures such as water and salts to form a gel. Glycosaminoglycan (GAG) is a proteoglycan that stores most of the water in the skin (Costin and Hearing, 2007; Bedoux et al., 2014). Water is essential for the skin to function properly. The influence of external factors can promote skin aging and skin aging destroys its barrier function. This makes it more fragile, and it gradually loses its natural elasticity and moisturizing function (Pimentel et al., 2017). Keratinocytes in the skin’s epidermis contain natural moisturizing factors (NMF), which are natural hygroscopic compounds, such as urea, polysaccharides, amino acids, and minerals (Brunt and Burgess, 2018; Pimentel et al., 2017). The lipids between the cells of the skin’s cuticle are also moisturizing, lining up to form a barrier to prevent water loss (Verdier-Sévrain and Bonté, 2007).

Marine organisms produce moisturizing molecules such as fatty acids and polysaccharides, which are commonly used in cosmetics. Algae-derived omega-6 polyunsaturated fatty acids, especially C-18 linoleic acid and gamma-linolenic acid, such as those found in marine microalgae, can be added to the oil in water emulsions to moisturize skin (Guillerme et al., 2017). A lack of unsaturated fatty acids has been reported to cause dermatitis and skin dehydration (Ziboh et al., 2000; Kim et al., 2008). Marine micro-organisms are important producers of unsaturated fatty acids. A strain of *Vibrio cyclitrophicus* isolated from the ocean has been reported to produce eicosapentaenoic acid (EPA; Abd Elrazak et al., 2013). According to Kim et al. (2008), *Nanochloropsis* sp. can produce EPA. *Cladophora glomerata*, a filamentous green alga of marine origin, was described as containing saturated fatty acids (C16:0) and unsaturated fatty acids (C16:1) (N-7) and (C18:1) (N-3), which can be used as an active moisturizing agent to prevent skin moisture loss (Couteau and Coiffard, 2020). The thickness of the cell wall of algae and the mucus layer formed by polysaccharides contained in the cell wall explain how they keep the cells hydrated (Stoyneva-Gärtner et al., 2020). Polysaccharides and oligosaccharides can be hydrogen-bonded to keratin to retain moisture (Bedoux et al., 2014). Wang et al. (2013) tested the moisturizing ability of polysaccharides extracted from five kinds of algae and found that polysaccharide DL extracted from Phaeophyceae had a good moisturizing effect, and its moisturizing performance was better than that of hyaluronic acid (HA). This indicated that seaweed polysaccharides could be used as an additive for moisturizing cosmetics. *Nostoc commune* moisturizing serum can be used to moisturize, whiten, and be non-greasy for the skin (Stoyneva-Gärtner et al., 2020).

In addition to the moisturizing function of seaweed polysaccharides, the extracellular polysaccharide (EPS) of marine bacteria also has moisturizing potential. The EPS of the *Polaribacter* sp. SM1127, isolated from Arctic kelp, had a good moisturizing ability that was superior to HA in cosmetics. This EPS has a significant protective effect on human dermal fibroblasts at low temperatures and can be used as a moisturizing ingredient in cosmetics (Sun et al., 2015). *Phyllobacterium* sp. 921F can produce a large amount of EPSs, and its water absorption and retention ability are better than collagen, chitosan, and glycerol (Li et al., 2015). There are few examples of bacterial exopolysaccharides used in cosmetics, so discovering these exopolysaccharides provides new moisturizing molecules for cosmetic formulations (Brunt and Burgess, 2018). A new strain of *Pseudoalteromonas* sp. has been isolated from polar regions. Intracellular extracts of the strains of RefirMAR® by BIOALVO have been applied to RefirMAR®, a cosmetic that is a good hydration agent (Martins et al., 2014). EPS HYD657, extracted *via* the fermentation of *Alteromonas macleodii* subsp. fijiensis biovar deepsane, is used in Abyssine® cosmetics (Martins et al., 2014). *Chlorella* extracts have been described to promote collagen synthesis and reduce wrinkles and appear to have value for skin-care products (Wang et al., 2015).

## Antiaging Effect

The aging process of the skin is affected by both internal factors and the external environment (Ganceviciene et al., 2012). Internal factor changes are mainly related to age, while external environmental stressors include high temperature, smoke, pollutants, and UV radiation (Zhang and Duan, 2018). Skin aging is mainly due to a reduction in collagen, elastic fibers, and hyaluronic acid and is manifested by wrinkles, dryness, loss of elasticity, sagging, and rough appearance (Brunt and Burgess, 2018). In the aging process of the skin, cells suffer from oxidative stress and lose the ability to regulate ROS (Bedoux et al., 2014). Fortunately, many substances from marine organisms effectively combat oxidative damage to cells and prevent skin aging. Fucoxanthin extracted from brown seaweed has been shown to protect keratinocytes from oxidative damage (Zheng et al., 2013). A glycosaminoglycan extracellular polysaccharide (HE 800) was discovered from the deep-sea bacteria *Vibrio diabolicus*, which can facilitate skin regeneration (Courtois et al., 2014). *Alteromonas* fermentation extract is an EPS produced by extremophiles living in deep-sea hydrothermal vents. The fermentation extracts of *Alteromonas* can reduce MDA (the end product of lipid peroxidation), chelate cadmium, and lead and form a protective film on the body surface (Borel et al., 2017). It was combined with carnosine, a sodium hyaluronate cross-polymer, and a tripeptide to create a new beauty cream, and the product was shown to improve facial contours and skin quality (Garre et al., 2017). This formulation has good tolerance and is an excellent antiaging product.

Marine micro-organisms contain many high-quality functional proteins and bioactive natural peptides with diverse molecular structures (Xia et al., 2021). For example, marine peptides derived from proteolytic products of *Navicula salinicola* (formerly *Navicula incerta*; Bacillariophyta) microalgae inhibit the activity of gamma-glutamate transferase (GGT), thereby reducing oxidative damage and delaying cell senescence (Kang et al., 2012). Similarly, peptides from *Chlorella* can reduce the expression of matrix metalloproteinase-1 (MMP-1) in human skin fibroblasts, thereby reducing the breakdown of collagen and delaying aging (Chen et al., 2011). We can use biotechnology to find more of these products from the ocean. A typical example of this is the research by Said Hassane et al. (2020), who studied microbial diversity from the marine sponge *Scopalina hapalia* using metagenomics and found that the microbial secondary metabolites had biological activity against seven targets associated with cell senescence. These were elastase, tyrosinase, catalase, sirtuin 1, cyclin-dependent kinase 7 (CDK7), fyn kinase, and proteasome. These data showed the potential of marine microbes to produce antiaging compounds.

## Repair Function

**Figure 4:**
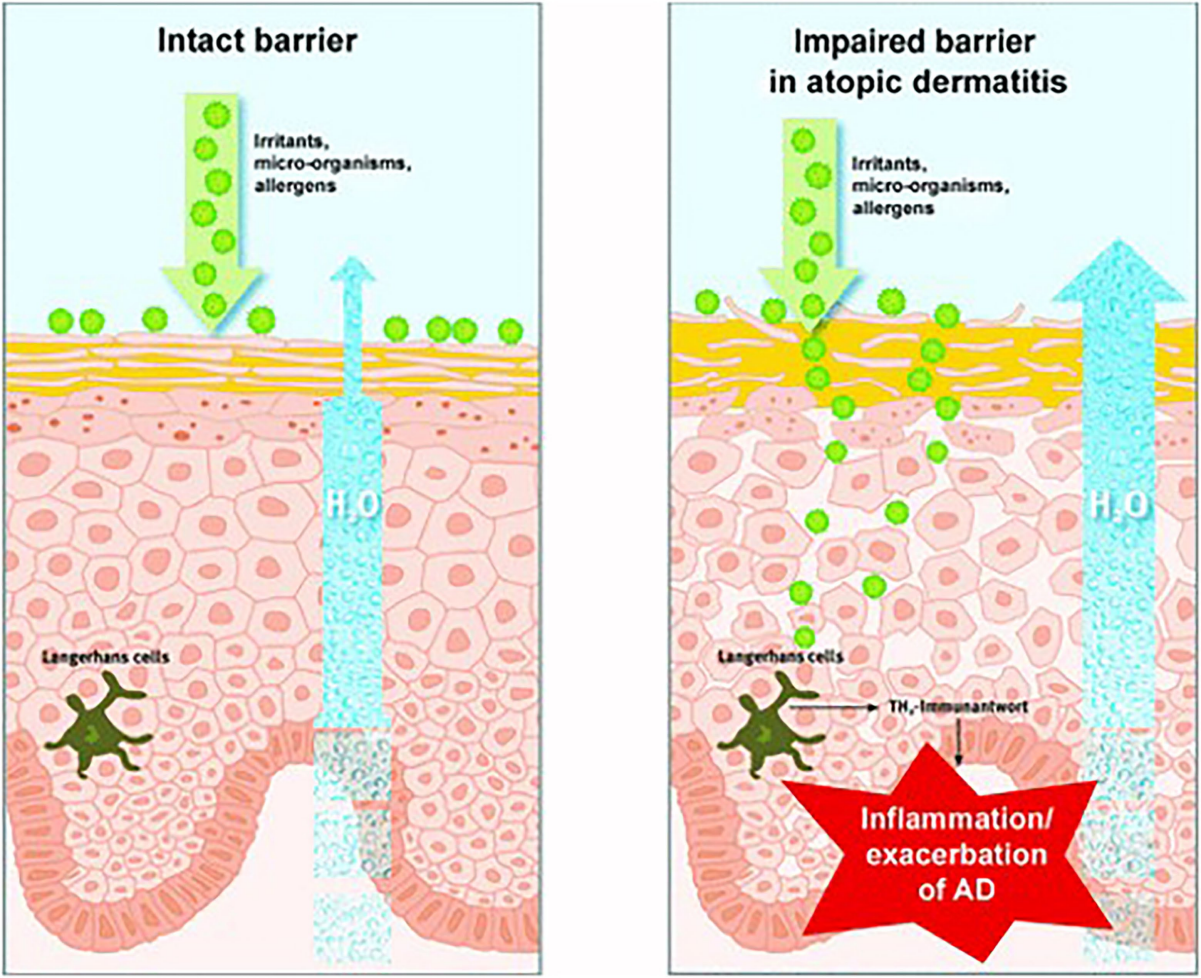
Reprinted with the kind permission of Journal der Deutschen Dermatologischen Gesellschaft publications (Proksch et al., 2009). The skin barrier is normal (left) and impaired (right). When the skin barrier is compromised, water loss increases, and environmental toxins can penetrate the skin, triggering irritation, allergic reactions, inflammation, or exacerbation of specific eczema.

The skin is the first barrier of the immune system, and its most important function is to separate the internal environment from the external environment. The skin barrier inhibits water loss and prevents harmful substances from entering (Proksch et al., 2009). The skin barrier mainly includes the cuticle, which contains protein-rich cells, keratinocytes, and lipid-rich cells. These lipids include cholesterol, free fatty acids, and ceramides (sphingolipids; Elias and Friend, 1975). When the skin barrier is damaged, the skin becomes dry and dehydrated. As a result, it is more easily invaded by external germs and irritants, which trigger inflammatory reactions and other symptoms (Proksch et al., 2009). Thus, skin repair is an important part of beauty care (Figure 4).

To deal with these problems, first, we can promote wound repair, that is, the growth or protection of skin fibrocytes. Zhang (2019) found that the EPS of marine bacteria *Polaribacter* sp. SM1127 can increase fibrocytes, promote the healing of skin wounds in mice, and reduce skin injury caused by low temperatures. In addition, the EPS of *Polaribacter* sp. SM1127 can also reduce lactate dehydrogenase (LDH) and ROS, increase glutathione (GSH) content, reduce superoxide dismutase (SOD) enzyme activity, and maintain cell activity and integrity to resist UV radiation damage. Letsiou et al. (2020) isolated *Aspergillus chevalieri* TC2-S6 from sponges (*Axinella*) and cultured it in potato dextrose broth (PDB) to obtain a component named ACCB from the culture medium. ACCB is mainly tetrahydroauroglaucin and flavoglaucin, and is capable of protecting human fibroblasts under oxidative stress conditions.

Second, we can protect the keratinocytes from damage by moisturizing. Long et al. (2010) cloned a new endo-type β-agarase gene agaA from the marine bacteria *Agarivorans* sp. LQ48 and expressed it in *E. coli*. This agaA enzyme (AgaA) had strong acid and alkali resistance. AgaA can produce DP6 (neoagarohexaose) and DP4 (neoagarotetraose) by hydrolysis from agarose, and these can be used as skin moisturizers. Collagen is a structural skin protein commonly used as the active ingredient in moisturizers (Brunt and Burgess, 2018). Swatschek et al. (2002) extracted collagen from kidney-shaped cartilage sponges and compared the effects of sponge-derived collagen with existing collagen in human skin. Marine collagen increased skin lipids, although both had similar moisturizing effects. We believe that marine collagen has a superior repair function because the added lipids help the skin hold moisture better.

In addition, we can also use substances with antioxidant effects to protect and repair skin cells, strengthen the vitality and connectivity of skin cells, enhance their immune capacity, and reduce free radical damage to skin cells. Squalene is an antioxidant, and hydrogenated squalane is used in cosmetics (Stoyneva-Gärtner et al., 2020). Squalene can also be added to moisturizers as an emollient that is quickly absorbed by the skin (Stoyneva-Gärtner et al., 2020). The HS-399 strain of *Aurantiochytrium acetophilum* sp. (a thraustochytrid) was isolated from a mangrove swamp in Biscayne Bay, Florida, United States, producing squalene and lipids (Ganuza et al., 2019). Moreover, bioactive indole derivatives were isolated from the marine sponge *Rhopaloeides odorabile* and its derived fungus *Hyrtios* sp., and DPPH (a stable free radical) was used to test its antioxidant capacity (Longeon et al., 2011); its antioxidant capacity was similar to that of Trolox. Compound 9 in the indole derivatives had no cytotoxicity and was suitable for skin repair. Raveendran et al. (2013) extracted mauran (MR), a highly polyanionic sulfated EPS from *Halomonas maura*, and showed that MR induces antioxidant properties by preventing the production of LPO (lipid peroxidation) and free radicals. It does not affect the production and function of the body’s own antioxidants, such as GSH (which normally functions as an antioxidant in cells), GR (which is essential for GSH production), and GPx (glutathione peroxidase). Li et al. (2011) isolated an aromatic polyketone compound from the sponge-derived fungus *Aspergillus versicolor*. Through the control experiment using standard antioxidants, including butylated hydroxyanisole (BHA), butylated hydroxytoluene (BHT), tertiarybutylhydroquinone (TBHQ), and ascorbic acid (VC), its excellent antioxidant performance was proven. On the other hand, lipase can also be used to produce whitening antioxidant products. For example, Sang (2017) screened microbial strains producing ferulic acid esterase (FAES1) from seawater. Ferulic acid is a phenolic acid with a strong antioxidant capacity and scavenging effect on free radicals. In addition, skin fibroblasts can be protected by enhancing lipid cell differentiation and promoting adiponectin synthesis, such as docosahexaenoic acid (DHA). DHA is usually obtained from marine fish oil (Lee et al., 2020), but due to environmental pollution, fish oil-derived DHA has become unreliable, and marine microbial DHA, produced by *Thraustochytrium* sp. 26185, can be used as a substitute. Meesapyodsuk and Qiu (2016) provided a partial solution to the biosynthesis mechanism of DHA. DHA has moisturizing and antiaging functions as well as tanning under certain conditions (Martini, 2017).

The above ideas provide a method for skin repair, but how to effectively combine these functional molecules to produce new skin-repair products still needs to be supported by experiments.

## Skin Lipid Conditioning

**Figure 5:**
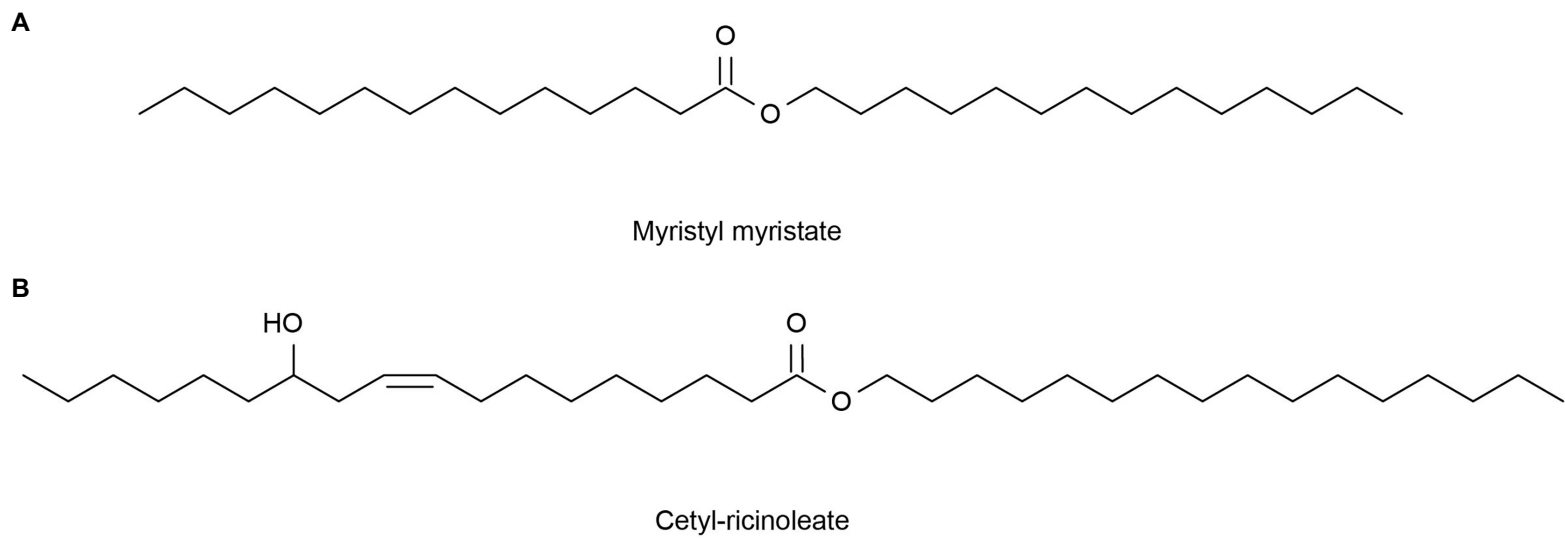
Reprinted with the kind permission of Applied Microbiology and Biotechnology publications (Metzger and Bornscheuer, 2006). Chemical structures of Myristyl myristate **(A)** and Cetyl-ricinoleate **(B)** are produced by lipase-catalyzed esterification.

The average rate of sebum production in normal human skin is about 1 mg/10 cm^2^ every 3 h (Hong et al., 2020). If the sebum secretion of human skin exceeds 1.5 mg/10 cm^2^ every 3 h, caused by high secretion of the sebaceous glands, then the skin becomes oily. However, a sebum secretion rate as low as 0.5 mg/10 cm^2^ per 3 h in human skin will result in dry skin (Endly and Miller, 2017). Excessive oil secretion may affect the microbial environment of the face, leading to acne (Youn, 2010). In addition, this can lead to enlarged facial pores and a greasy facial appearance (Hong et al., 2020). Therefore, it is particularly important to clean the face. Lipase can catalyze the hydrolysis of insoluble triglycerides into glycerol diesters and glycerol monomers (Yvergnaux, 2017) to produce cleansing facial oil. Compared with chemical reagents, the enzymes are milder and less irritating, and many of these commercial products now have enzymes added (Liu et al., 2016). Lipase comes from a variety of sources, including animals, plants, and microorganisms. Significantly, microorganisms have many advantages, such as easy cultivation, short generation cycles, and resistance to external environmental conditions (Hasan et al., 2006). Marine micro-organisms can generate many lipases that can withstand various conditions due to their unique environment, better meeting the needs of commercial production and practice requirements, so they are favored by people. For example, Su et al. (2015) obtained alkaline-resistant lipase (LipA) from a metagenomic library of the sponge *Ircinia* sp. Yuan et al. (2016) expressed a MAS1 gene derived from the marine *Streptomyces* strain W007 in *Pichia pastoris* and proved that MAS1 is a heat-resistant and alkali-resistant lipase. Nishihara et al. (2008) isolated a bacterium HFKI0020 from the intestinal contents of marine fish that could produce phospholipase A1 at low temperatures. The skin barrier of patients with dry skin tends to be itchy, red in color, scaly, and easily injured (Tončić et al., 2018). Lipase can be used to produce simple lipids for dry skin tonings, such as myristyl myristate (Figure 5A) and cetyl-ricinoleate (Figure 5B; Metzger and Bornscheuer, 2006). In addition, collagen is not absorbed by the skin, but binds to water through hydration and attaches to the skin, thereby maintaining moisture (Swatschek et al., 2002). Swatschek et al. (2002) collected the *Chondrosia reniformis* Nardo sponge from the Aegean Sea and extracted collagen, proving that this sea-derived collagen has a good moisturizing ability and can increase skin lipids. These lipids can treat dry skin instead of traditional collagen products.

## Aromatic Substances

**Figure 6:**
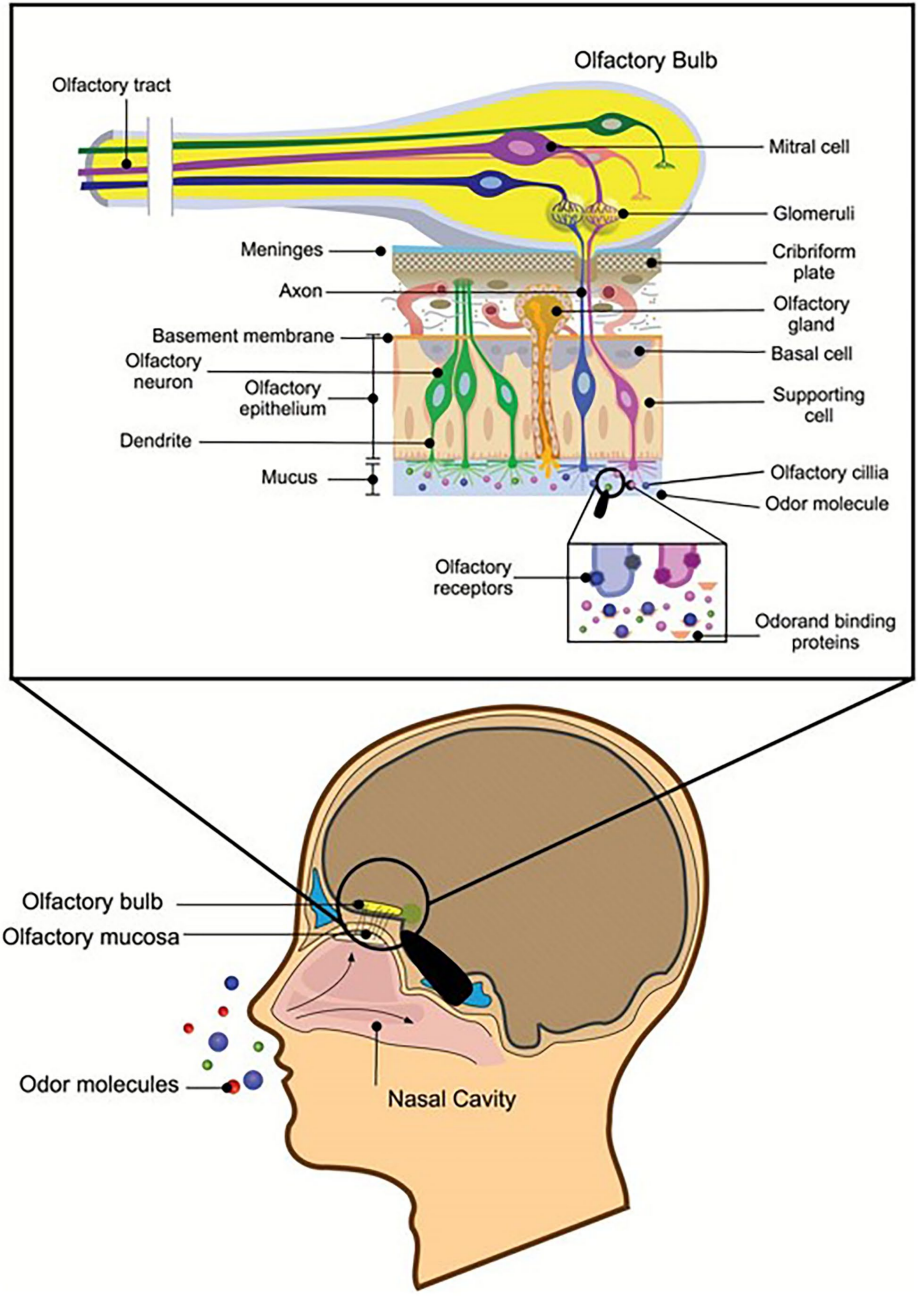
Reprinted with the kind permission of Chemical Senses publications (Silva Teixeira et al., 2015). Schematic representation of a sagittal section through the human head with a section of the olfactory nervous system depicted in greater detail. Air transmits odor molecules to olfactory mucosa, combines with odor transporter proteins to form complexes, and transports them to olfactory receptors on olfactory cilia. After a series of signal transductions, they are transmitted to the olfactory tract to produce olfactory sensations.

Fragrances are aromatic substances that can be perceived by smell. Olfactory cells on the mucous membrane inside the nasal cavity can accept the stimulation of odor molecules and produce olfactory stimuli. The smell of perfume passes the nasal cavity and stimulates the olfactory cell, the olfactory center that transmits the fragrance to the cerebrum, thereby forming olfactory sensations and bringing joyful experiences (Silva Teixeira et al., 2015). Fragrances are generally short-chain fatty acids or alcohol esters (Macedo et al., 2003), that are pleasing to people and are often used in cosmetic products. On the one hand, the natural volatile aromas of some marine microbes could be used as a new source of perfume; on the other hand, known perfume ingredients can be produced through biological transformation. These methods are greener and more environmentally friendly than those used in the traditional chemical industry. Most compounds with volatile fragrances belong to terpenes (Riad et al., 2021). *Synechococcus* PCC 6911 can reduce Geranial (1) into Geraniol (2), and *Synechococcus* PCC 6716 transforms (−)-Menthone ((−)-4) into (−)-Menthol ((−)-5), which are important components of perfumes’ essential oils (Jϋttner and Hans, 1986). These needed spices can also be produced by the catalysis of natural enzymes, such as acetic acid and cinnamic acid. Wang (2016) screened lipase L-1, which can synthesize cinnamic acid acetate with the highest conversion rate from the genome of the deep-sea micro-organism *Streptomyces* sp. SCSIO 13580. Recombinant enzymes designed for specific substrates are more efficient than traditional tools for biotransformations, such as micro-organisms. Fu et al. (2012) achieved recombinant expression of ene-reductase from *Synechococcus* sp. PCC 7942 and obtained a new ene-reductase that can efficiently convert (*R*)-(−)-carvone to (2*R*, 5*R*)-dihydrocarvone. Additional aromas from marine microbes are likely to be discovered in the future (Figure 6).

## Pigment Substances

Beautiful colors provide sensory enjoyment and are found in nail polish, lipstick, and other cosmetics. Industrial pigments are usually synthesized from benzene, toluene, and other chemical reagents, so they can be toxic and unacceptable to many people (Kalra et al., 2020). Natural pigments are widely used in products due to their safety and stability. They are represented by anthocyanins, carotenoids, and chlorophyll (Kalra et al., 2020). Marine microbes are considered sources of inexpensive, novel, stable, and safe biological pigments, so many relevant studies have been reported. Du et al. (2008) obtained two pigments from *Aspergillus glaucus* isolated from marine sediments around mangrove roots in Fujian Province, China. These were named yellow pigment (+) variecolorquinones A and red pigment aspergiolide B. Dhale and Vijay-Raj (2009) isolated *Penicillium* sp. NIOM-02 from marine sediments in Miramar (India). The removal of the DPPH radical and the production of red dye were achieved simultaneously. When it was cultured on malt extract agar (MEA) plates, the red pigment secreted by the fungus occurred around its colony. This showed that the red dye has good water solubility. *Penicillium bilaii* (MST-MF667) was isolated from the Huon Estuary, Tasmania, and subjected to chemical analysis. Analysis revealed two yellow pigments including (−)-2, 3-dihydrocitromycetin and (−)-2, 3-dihydrocitromycin (Capon et al., 2007). Ganesh Kumar et al. (2013) isolated the hMGM-7 [MTCC 11712] strain from the surface of *Hypnea musciformis* (Rhodophyta) and found that it could produce acid-resistant melanin.

In addition, some invertebrates in the ocean, such as sponges and corals, are very colorful and these bright colors may be related to the photosynthetic pigments of symbiotic micro-organisms. Xu et al. (2008) isolated *Aspergillus* sp. from coral reefs in Manado, Indonesia, and obtained the red pigment bostrycin and two new yellow hexahydroanthrones, named tetrahydrobostrycin and 1-deoxytetrahydrobostrycin. Yellow compounds were isolated from *Eurotium cristatum*, a fungus from a sponge *Mycale* sp., which contains 2-(2′, 3-epoxy-1′, 3′-heptadienyl)-6-hydroxy-5-(3-methyl-2-butenyl) benzaldehyde and 1, 8-dihydroxy-6-methoxy-3-methyl-9, 10-anthracenedione (physcion). Phycocyanin produced by thermophilic cyanobacteria can be used to make eye shadows (Bermejo et al., 2003). Phycocyanin from *Spirulina* has been used as a colorant in eye shadow by Ink Chemicals in Japan (Ryu et al., 2015; Pangestuti et al., 2020; Yarkent et al., 2020). Pink and purple pigments in cosmetics can also be formulated from natural pigments extracted from red microalgae (Arad and Yaron, 1992). Biotechnically, obtained R-phycoerythrin has been used in colored creams and cosmetics as a natural dye instead (Bedoux et al., 2014). Antioxidant phycoerythrobilins from *Arthrospira/Spirulina* (Cyanobacteria) and *Porphyridium* (Rhodophyta) can be used in lipstick and eyeliner (Hamed, 2016). In summary, pigments derived from marine micro-organisms are now widely used in the cosmetics industry.

## Others

The size and largely unexplored nature of the ocean often produce surprising discoveries. The complex and diverse ocean environment leads to metabolic pathways and adaptation mechanisms of marine micro-organisms that are completely different from those of terrestrial organisms. These pathways generate various natural products with unique structures, great diversity, and significant biological activities (Zhang et al., 2021). Jin et al. (2020) used AGAR biodegradation bacteria *Flammeovirga pacifica* WPAGA1 isolated from deep-sea locations to prepare algal oligosaccharide (AOS). AOS inhibited dihydrotrophil (DHT) to prolong the hair growth period of mice and might be used to prevent androgenetic alopecia (AGA). Peng et al. (2006) isolated the bacterial strain A4B-17 belonging to microspheres from sea squirts in the coastal waters of Palau. A4B-17 can generate alkyl esters of 4HBA (4-hydroxybenzoate), which can effectively prevent the growth of yeast, mold, and Gram-positive bacteria. This indicated it could possibly be used as a cosmetic preservative. The compounds extracted from microalgae can be used as the main components of cosmetics, but they can also be used as cosmetic stabilizers, dyes, thickeners, and other auxiliary materials depending on their different characteristics (Ryu et al., 2015; Wang et al., 2015). The rich pigments of microalgae can also be used in deodorants, antioxidants, creams, cleansers, and other products. They can also be used as deodorants because of their ability to mask odors. Vitamins produced by green unicellular *Chlorella* can promote hair growth by treating dandruff (Stoyneva-Gärtner et al., 2020). Seaweed polysaccharides are also widely used. Cationic polysaccharides derived from seaweed, such as chitosan, are very useful film-forming agents and are widely used in hair damage care and gel fixing products due to their unique advantage of binding tightly to the proteins (negative charges) found on human skin and hair (Kanlayavattanakul and Lourith, 2015). Chitosan can be used in dental products, such as toothpaste and chewing gum (Rinaudo, 2006). Non-ionic polysaccharides such as hydroxymethylcellulose can be used in nail products such as film formers and thickeners (Kanlayavattanakul and Lourith, 2015). Polysaccharides extracted from algae, such as D-glucose, D-mannose, D-galactose, and D-glucuronic acid, have been considered excipients in cosmetics due to their viscosity (Kim et al., 2008). Seaweed-derived tocopherol can be used in baby wipes, eyebrow growth serums, beard creams, and hair shampoo (Stoyneva-Gärtner et al., 2020). These versatile and strange natural products draw the scientist’s mind. The ocean’s treasures are almost endless, and they are waiting to be discovered.

## Conclusion

This review covers the recent research and applications of natural products from marine micro-organisms in cosmetics, including marine bacteria, fungi, microalgae, and other micro-organisms. Starting from functional aspects, the mechanism and potential of natural compounds such as phenols, polysaccharides, vitamins, enzymes, proteins, and peptides in the field of cosmetics are summarized. As mentioned above, naturally active substances extracted from marine microbes have fewer skin side effects than chemically synthesized substances and can reduce skin damage while maintaining beauty. However, the exact mechanisms by which most of these natural compounds act on the skin have not been fully studied. We believe that it is necessary to further study these compounds to determine their mechanisms of action, preferably through clinical trials or *in vitro* cell tests to investigate the absorption and sensitization of these active substances on different skin types. This not only helps to improve the quality of cosmetics but also helps different groups of people to better choose cosmetics to protect the rights and interests of consumers and promote the benign development of the cosmetics market.

However, relatively little development of marine resources has been made to date. The unique environment of the ocean produces rich microbial resources and includes marine sediments, the symbiotic micro-organisms of invertebrates, and the intestinal micro-organisms of marine fish. The environment of these micro-organisms is very different from that of terrestrial organisms, endowing them with unique metabolic pathways and adaptation mechanisms. Adaptation to the marine environment also makes organisms unable to survive outside of this environment and difficult to culture. This is a great obstacle to developing and utilizing new molecules and enzymes, but metagenomic technology may help in this regard. Metagenomics can extract DNA from the whole environment to obtain the genome of unculturable micro-organisms in the whole environment and has been considered a good method to utilize the natural active substances of unculturable micro-organisms (Martins et al., 2014). Said Hassane et al. (2020) used metagenomic technology to discover metabolites with antiaging potential from microorganisms in marine sponges. Su et al. (2015) obtained LipA from a metagenomic library of the sponge *Ircinia* sp. Yuan et al. (2016) expressed the MAS1 gene derived from marine *Streptomyces* strain W007 in *P. pastoris* and demonstrated that MAS1 is a heat-resistant and alkali-resistant lipase. These results provide us with ideas for the exploitation of marine microbial resources. In addition, we know that most of the natural products of marine micro-organisms have multiple functions. For example, collagen not only has the functions of antioxidation and anti-UV radiation but also has a moisturizing function. The combination of these functions provides it with an antiaging function (Swatschek et al., 2002). Moreover, using marine species to produce more green and environmentally friendly products through biotransformation has become a new choice for cosmetic manufacturers. In developing these types of materials in cosmetics, we can combine a variety of non-interfering substances to produce greater functionality. These active substances produced by marine microbes will be brought to market safely and effectively.

## Author Contributions

JD and BW are responsible for writing the whole passage. LC is responsible for giving suggestions on revision and review. All authors contributed to the article and approved the submitted version.

## Funding

This work was supported by Fuzhou University Testing Fund of precious apparatus (Grant/Award Number: 2022T040), Natural Science Foundation of Fujian Province (Grant/Award Number: 2021Y0094, 2021Y0095, 2021J01607), Fujian Provincial Clinical Research Center for Cancer Radiotherapy and Immunotherapy (Grant/Award Number: 2020Y2012), Joint Funds for the innovation of science and Technology, Fujian province (Grant/Award Number: 2018Y9105), Sponsored by Fuzhou health technology project (Grant/Award Number: 2021S263).

## Conflict of Interest

The authors declare that the research was conducted in the absence of any commercial or financial relationships that could be construed as a potential conflict of interest.

## Publisher’s Note

All claims expressed in this article are solely those of the authors and do not necessarily represent those of their affiliated organizations, or those of the publisher, the editors and the reviewers. Any product that may be evaluated in this article, or claim that may be made by its manufacturer, is not guaranteed or endorsed by the publisher.

## Supplementary Material

The Supplementary Material for this article can be found online at: https://www.frontiersin.org/articles/10.3389/fmicb.2022.892505/full#supplementary-material
