# Supplementary material for: Application of Marine Microbial Natural Products in Cosmetics
Source: Front Microbiol. 2022 May 26;13:892505. doi: 10.3389/fmicb.2022.892505 (PMC9196241; doi:10.3389/fmicb.2022.892505)
Supplement: Supplementary file 1 [file Table_1.DOCX]

| Table 1. Potential function of natural products of marine micro-organisms in cosmetics |
| --- |

| Compound | Source | Function | Habitat | References |
| --- | --- | --- | --- | --- |
| mycosporine-like amino acids | gene clusters in *Actinosynnema mirum* DSM 43827 and *Pseudonocardia* P1. | anti-UV | marine | **Miyamoto et al.** **(2014)** |
| astaxanthin | *Haematococcus pluvialis, Phaffia*, *Rhodozyma*, *Xanthophyllomyces, Nannochloropsis* sp. *Haematococcus* sp. | antioxidant, tyrosinase inhibitors | marine | **Naguib. (2000),** **Ambati et al. (2014), Balasubramaniam et al. (2021)** |
| lycopene | *Anabaena* | photoprotection | marine | **Hashtroudi et al. (2013)** |
| β-carotene | *Anabaena*, *Nostoc* cyanobacteria | photoprotection | marine | **Mezzomo and Ferreira. (2016)** |
| fucoxanthin | Microalgal, brown seaweed | against sunburn, antioxidant | marine | **Matsui et al. (2016), Zheng et al. (2013)** |
| (3*R*, 2’*S*)-myxol | Flavobacteriaceae | antioxidant | marine | **Shindo et al. (2007)** |
| (3*R*)-saproxanthin | Flavobacteriaceae | antioxidant | marine | **Shindo et al. (2007)** |
| cyclosporine I | *Exophiala* | anti-UVA | marine fungus | **Zhang et al. (2008)** |
| golmaenone of diketopiperazine alkaloid | *Aspergillus* sp. | anti-UV | marine fungus | **Li et al. (2004)** |
| neoechinulin A of related alkaloids | *Aspergillus* sp. | anti-UV | marine fungus | **Li et al. (2004)** |
| RRR-α-tocopherol | *Stichococcus bacillaris* strain siva2011 | antioxidant | marine | **Sivakumar et al. (2014)** |
| α-tocopherol | *Raphidonema* | antioxidant | snow and permafrost substrates | **Leya et al. (2009)** |
| Kojic acid | *Altenaria* sp. | tyrosinase inhibitors | marine algae | **Li et al. (2003)** |
| Myrothenones A | *Myrothecium* sp. strain MFA58 | tyrosinase inhibitors | marine algae | **Li et al. (2005)** |
| Homothallin II | *Trichoderma* sp. | tyrosinase inhibitors | marine | **Tsuchiya et al. (2008)** |
| 1β,5α,6α,14-tetraacetoxy-9α-benzoyloxy-7βH-eudesman-2β,11-diol and 4α,5α-diacetoxy-9α-benzoyloxy-7βH-eudesman-1β,2β,11, 14-tetraol | *Pestalotiopsis* sp. | tyrosinase inhibitors | marine | **Wu et al. (2013)** |
| N-acyl dehydrotyrosine derivatives | *Thalassotalea* sp. strain PP2-459 | tyrosinase inhibitors | marine crustaceans | **Deering et al. (2016)** |
| methylene chloride | *Pseudomonas* sp. | tyrosinase inhibitors | sandbar | **Kang et al. (2011)** |
| (-)-4-hydroxysattabacin | *Bacillus* sp. | tyrosinase inhibitors | marine sediments | **Kim et al. (2017)** |
| cydromicin(1) | *Tolypocladium* sp. | tyrosinase inhibitors | arctic glacial sediments | **Khan et al. (2021)** |
| pseudoalteromone A (1) | *Pseudoalteromonas* sp*.* | tyrosinase inhibitors | marine | **Lim et al. (2021)** |
| eicosapentaenoic acid (EPA) | *Vibrio cyclitrophicus, Nanochloropsis* sp. | moisturizing | marine | **Abd Elrazak et al. (2013), Kim et al.** **(2008)** |
| saturated fatty acids (C16:0) and unsaturated fatty acids (C16:1) (N-7) and (C18:1) (N-3) | *Cladophora glomerata* | moisturizing | filamentous green microalga of marine origin | **Couteau and Coiffard. (2020)** |
| polysaccharide DL | *Phaeophyta* | moisturizing | marine | **Wang et al. (2013)** |
| extracellular polysaccharide (EPS) | *Polaribacter* SM1127 | moisturizing | arctic kelp | **Sun et al. (2015)** |
| EPS HYD657 | *Alteromonas macleodii* subsp*.* | moisturizing | marine | **Martins et al. (2014)** |
| EPS | *Alteromonas* | antiaging | deep-sea hydrothermal vents | **Borel et al. (2017)** |
| intracellular extracts of the strains | *Pseudoalteromonas* sp. | hydration | polar regions | **Martins et al. (2014)** |
| glycosaminoglycan EPS (HE 800) | *Vibrio diabolicus* | facilitate skin regeneration | deep-sea | **Courtois et al. (2014)** |
| EPS | *Polaribacter* sp. SM1127 | promote wound repair, anti-UV | marine | **Zhang (2019)** |
| ACCB | *Aspergillus chevalieri* TC2-S6 | protecting human fibroblasts | sponges (*Axinella*) | **Letsiou et al. (2020)** |
| agaA enzyme | *Agarivorans* sp. LQ48 | produces moisturizing molecules | marine | **Long et al. (2010)** |
| collagen | kidney-shaped cartilage sponges, *Chondrosia reniformis* Nardo sponge | moisturizing, increasing skin lipids | marine | **Swatschek et al. (2002)** |
| squalene | *Aurantiochytrium acetophilum* sp. HS-399 | antioxidant, emollient | mangrove swamp in Biscayne Bay, Florida, USA | **Ganuza et al. (2019)** |
| bioactive indole derivatives | *Rhopaloeides odorabile*, *Hyrtios* sp. | antioxidant | marine sponge and fungus | **(Longeon et al. 2011)** |
| mauran (MR) | *Halomonas maura* | antioxidant | marine | **Raveendran et al. (2013)** |
| aromatic polyketone compound | *Aspergillus versicolor* | antioxidant | marine | **Li et al. (2011)** |
| alkaline resistant lipase (LipA) | *Ircinia* sp*.* | cleaning facial grease | sponge | **Su et al. (2015)** |
| heat-resistant and alkali-resistant lipase | *Streptomyces* sp. | cleaning facial grease | marine | **Yuan et al. (2016)** |
| lipase L-1 | *Streptomyces* sp. SCSIO 13580 | catalyzed spice production | deep-sea | **Wang (2016)** |
| ene-reductase | *Synechococcus* sp. PCC 7942 | catalyzed spice production | marine sediments | **Fu et al. (2012)** |
